# Fecal Microbiota Transplantation Role in the Treatment of Alzheimer's Disease: A Systematic Review

**DOI:** 10.7759/cureus.29968

**Published:** 2022-10-06

**Authors:** Sondos T Nassar, Tasniem Tasha, Anjali Desai, Anjana Bajgain, Asna Ali, Chandrani Dutta, Khadija Pasha, Salomi Paul, Muhammad S Abbas, Sathish Venugopal

**Affiliations:** 1 Medicine and Surgery, California Institute of Behavioral Neurosciences & Psychology, Fairfield, USA; 2 Internal Medicine, California Institute of Behavioral Neurosciences & Psychology, Fairfield, USA; 3 Department of Psychology, California Institute of Behavioral Neurosciences & Psychology, Fairfield, USA; 4 Family Medicine, California Institute of Behavioral Neurosciences & Psychology, Fairfield, USA; 5 Pediatric, California Institute of Behavioral Neurosciences & Psychology, Fairfield, USA; 6 Medicine, California Institute of Behavioral Neurosciences & Psychology, Fairfield, USA

**Keywords:** short-chain fatty acids, alzheimer's disease, animal studies, memory issues, cognitive abilities, neurodegenerative disesase, gut microbiome, gut-brain connection, alzheimer’s dementia, fecal microbiota transplant

## Abstract

Alzheimer's, a neurodegenerative disease that starts slowly and worsens progressively, is the leading cause of dementia worldwide. Recent studies have linked the brain with the gut and its microbiota through the microbiota-gut-brain axis, opening the door for gut-modifying agents (e.g., prebiotics and probiotics) to influence our brain's cognitive function. This review aims to identify and summarize the effects of fecal microbiota transplantation (FMT) as a gut-microbiota-modifying agent on the progressive symptoms of Alzheimer's disease (AD). This systematic review is based on the Preferred Reporting Items for Systematic Reviews and Meta-Analyses (PRISMA) 2020 guidelines. A systematic search was done using Google Scholar, PubMed, PubMed Central, and ScienceDirect databases in June 2022. The predefined criteria upon which the studies were selected are English language, past 10 years of narrative reviews, observational studies, case reports, and animal studies involving Alzheimer's subjects as no previous meta-analysis or systematic reviews were done on this subject.

Later, a quality assessment was done using the available assessment tool based on each study type. The initial search generated 4,302 studies, yielding 13 studies to be included in the final selection: 1 cohort, 2 case reports, 2 animal studies, and 8 narrative reviews. Our results showed that FMT positively affected AD subjects (whether mice or humans). In humans, the FMT effect was measured by the Mini-Mental State Examination (MMSE), showing improvement in Alzheimer's symptoms of mood, memory, and cognition. However, randomized and nonrandomized clinical trials are essential for more conclusive results.

## Introduction and background

Alzheimer's disease 

Alzheimer's disease (AD) is the most prevalent dementia worldwide, affecting more than 45 million patients without a known cure yet [1]. The pathogenesis of AD starts with the brain accumulating damage over 15-20 years, such as synaptic and mitochondrial alterations, vessel injury, chronic neuroinflammation, cognitive dysfunction, and neuronal cell death affecting multiple systems, after which the clinical symptoms become overt [1]. AD's dominant histological lesions identified in the brain are amyloid-beta (Aβ) extracellular plaques and intracellular neurofibrillary tangles enriched with hyperphosphorylated Tau protein [1,2]. The Aβ-soluble oligomeric form is the most neurotoxic species and the key correlate of the disease gravity [2]. However, multiple anti-Aβ-targeting trials have failed as a route of treating AD; thus, perhaps, the complexity of its pathogenesis requires alternative and multitarget therapies [3].

The microbiota-gut-brain axis

**Figure 1:**
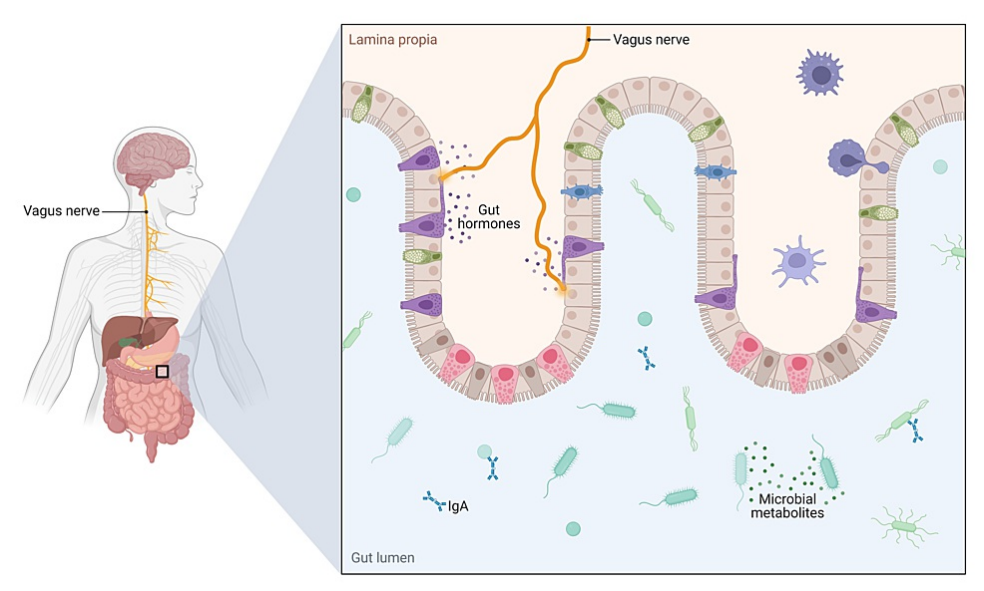
The microbiota-gut-brain axis. Figure credit: BioRender.com

The microbiota-gut-brain axis is an association between the microbiota, the gut, and the brain that synchronizes the gut with the CNS to modify behavior and the brain's immune homeostasis [4]. Microbial metabolites, including short-chain fatty acids (SCFAs), protein, and tryptophan metabolites; the immune system; the vagus nerve; and the enteric nervous system run the bidirectional communication between the gut and the brain (Figure 1) [4].

**Figure 2:**
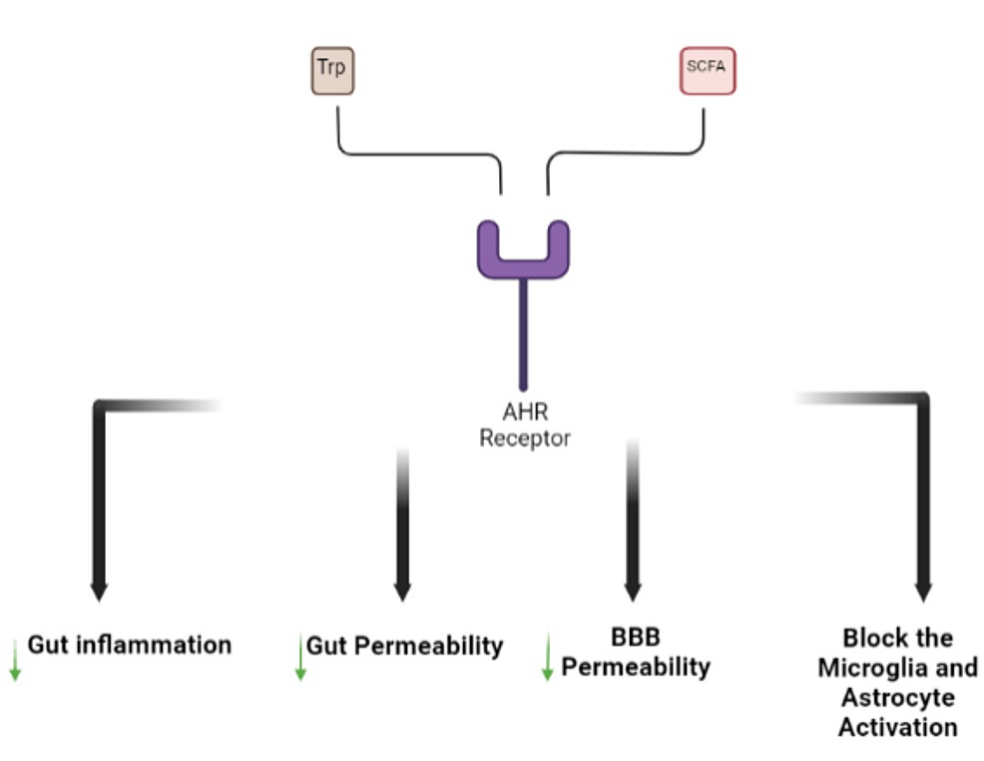
Ligands of the AHR receptor. AHR, aryl hydrocarbon receptor; SCFA, short-chain fatty acid; Trp, tryptophan; BBB, blood-brain barrier Figure credit: BioRender.com

This association explains the recent studies showing how the gut microbiota plays a vital role in neuropsychiatric [5,6] and neurodegenerative [7,8] disorders by metabolizing the soluble fibers, peptides, and proteins, and their main products are SCFAs. The gut microbiota also metabolizes the dietary proteins, bringing out tryptophan and other metabolites. The SCFAs and tryptophan metabolites are ligands of the aryl hydrocarbon receptor (AHR), and by activating this receptor, a decrease in gut inflammation, in addition to a reduction in the gut and blood-brain barrier (BBB) permeabilities and blockage of the microglia and astrocyte activation, is noticed to trigger the gut and brain homeostasis (Figure 2) [4].

Understandably, considerable shifts in human gut microbiota have been spotted in the CNS disorders such as anxiety, autism, and depression [5,6] and neurodegenerative diseases such as Alzheimer's and Parkinson's [7,8]. It has then been thought that the microbiome-modifying agents, e.g., probiotics and prebiotics, will improve some AD symptoms [9].

In this review, another microbiome-modifying method and its effect on AD symptoms and progression will be studied.

Fecal microbiota transplantation

Fecal microbiota transplantation (FMT) is the process of transferring fecal material from a fit donor to a receiver to calibrate the recipient's intestinal composition and, thus, the function of intestinal microbiota [10]. FMT has successfully treated many gastrointestinal disorders [11], mainly used to treat recurrent *Clostridium difficile *infections [12].

FMT has also been described as lessening intestinal dysbiosis, alleviating physical impairment in Parkinson's disease mice [13], and reducing alcohol-induced anxiety and depression in animal models [14,15].

Therefore, this review aims to focus on FMT's role in AD symptoms and progression, for it is about time we start looking for the potential involvement of the gastrointestinal microbiota in AD pathophysiology. This new linkage may provide worthy targets for new treatment approaches and modalities to delay the onset, slow the progression, or reverse the disease process, eventually reducing the ever-increasing prevalence of AD.

## Review

Method

This study is based on the Preferred Reporting Items for Systematic Reviews and Meta-Analyses (PRISMA) 2020 guidelines [16].​​​​​​

Eligibility Criteria

This review question was formulated based on the participants, intervention, and outcome (PIO) elements: participants, Alzheimer's patients, or AD mice models; intervention, fecal microbiota transplantation; and outcome, any change, whether for the better or the worse, of AD symptoms. Besides, additional inclusion and exclusion criteria were added. Inclusion criteria: English language, free full-text articles published within the past 10 years, human and animal studies, literature reviews, gray literature, randomized controlled trials (RCTs), observational studies, case reports, systematic reviews, and meta-analyses. Exclusion criteria: editorials and papers that were published in languages other than English.

Databases and Search Strategy

The search was conducted systematically using Google Scholar, PubMed, PubMed Central (PMC), and ScienceDirect databases. The last search of all the databases was conducted on June 2022. The key terms used in the search engines were AD, fecal microbiota transplantation, dementia, bacteriotherapy, and the Medical Subject Heading (MeSH) strategy used in PubMed. Details of the databases and search strategies can be found in Table 1.

**Table 1:** Detailed description of databases' search terms and results. PMC, PubMed Central

Databases	Keywords	Search strategy	Number of articles before filters	Filters	Search result
PubMed	Fecal microbiota transplantation, gut microbiota transplantation, fecal transplant, bacteriotherapy, stool transplant, Alzheimer’s, dementia, neurodegenerative disorder, memory loss disorder	("fecal microbiota transplantation"[MeSH Terms] OR ("fecal"[All Fields] AND "microbiota"[All Fields] AND "transplantation"[All Fields]) OR "fecal microbiota transplantation"[All Fields] OR (("gastrointestinal microbiome"[MeSH Terms] OR ("gastrointestinal"[All Fields] AND "microbiome"[All Fields]) OR "gastrointestinal microbiome"[All Fields] OR ("gut"[All Fields] AND "microbiota"[All Fields]) OR "gut microbiota"[All Fields]) AND ("transplantability"[All Fields] OR "transplantable"[All Fields] OR "transplantated"[All Fields] OR "transplantating"[All Fields] OR "transplantation"[MeSH Terms] OR "transplantation"[All Fields] OR "transplantations"[All Fields] OR "transplanted"[All Fields] OR "transplanting"[All Fields] OR "transplantation"[MeSH Subheading] OR "transplantation s"[All Fields] OR "transplanter"[All Fields] OR "transplanters"[All Fields] OR "transplantion"[All Fields] OR "transplants"[MeSH Terms] OR "transplants"[All Fields] OR "transplant"[All Fields])) OR ("fecal microbiota transplantation"[MeSH Terms] OR ("fecal"[All Fields] AND "microbiota"[All Fields] AND "transplantation"[All Fields]) OR "fecal microbiota transplantation"[All Fields] OR ("fecal"[All Fields] AND "transplant"[All Fields]) OR "fecal transplant"[All Fields]) OR ("bacteriotherapies"[All Fields] OR "bacteriotherapy"[All Fields]) OR ("fecal microbiota transplantation"[MeSH Terms] OR ("fecal"[All Fields] AND "microbiota"[All Fields] AND "transplantation"[All Fields]) OR "fecal microbiota transplantation"[All Fields] OR ("stool"[All Fields] AND "transplant"[All Fields]) OR "stool transplant"[All Fields]) OR ("fecal microbiota transplantation/pharmacology"[MeSH Terms] OR "fecal microbiota transplantation/psychology"[MeSH Terms] OR "fecal microbiota transplantation/statistics and numerical data"[MeSH Terms] OR "fecal microbiota transplantation/therapeutic use"[MeSH Terms] OR "fecal microbiota transplantation/therapy"[MeSH Terms])) AND ("alzheime s"[All Fields] OR "alzheimer disease"[MeSH Terms] OR ("alzheimer"[All Fields] AND "disease"[All Fields]) OR "alzheimer disease"[All Fields] OR "alzheimer"[All Fields] OR "alzheimers"[All Fields] OR "alzheimer s"[All Fields] OR "alzheimers s"[All Fields] OR ("dementia"[MeSH Terms] OR "dementia"[All Fields] OR "dementias"[All Fields] OR "dementia s"[All Fields]) OR ("neurodegenerative diseases"[MeSH Terms] OR ("neurodegenerative"[All Fields] AND "diseases"[All Fields]) OR "neurodegenerative diseases"[All Fields] OR ("neurodegenerative"[All Fields] AND "disorder"[All Fields]) OR "neurodegenerative disorder"[All Fields]) OR (("memory disorders"[MeSH Terms] OR ("memory"[All Fields] AND "disorders"[All Fields]) OR "memory disorders"[All Fields] OR ("memory"[All Fields] AND "loss"[All Fields]) OR "memory loss"[All Fields]) AND ("disease"[MeSH Terms] OR "disease"[All Fields] OR "disorder"[All Fields] OR "disorders"[All Fields] OR "disorder s"[All Fields] OR "disordes"[All Fields])) OR ("alzheimer disease/diet therapy"[MeSH Major Topic] OR "alzheimer disease/metabolism"[MeSH Major Topic] OR "alzheimer disease/microbiology"[MeSH Major Topic] OR "alzheimer disease/physiology"[MeSH Major Topic] OR "alzheimer disease/physiopathology"[MeSH Major Topic] OR "alzheimer disease/rehabilitation"[MeSH Major Topic]))	164	Free full text, from 2012 to 2022, English	93
PMC	Fecal microbiota transplantation, Alzheimer’s disease	Fecal microbiota transplantation AND Alzheimer's disease	1,594	10 years cutoff	1,588
Google Scholar	Fecal microbiota transplantation, bacteriotherapy, Alzheimer's, dementia	"Fecal Microbiota transplantation" OR "bacteriotherapy" AND "Alzheimer's" OR "Dementia"	2120	10 years cutoff	1,980
Science Direct	Fecal microbiota transplantation, Alzheimer's disease	"Fecal microbiota transplantation" AND "Alzheimer's disease"	664	10 years cutoff	641

All references were grouped and alphabetized using EndNote, and duplicate removal was done both by EndNote and manually. Then, the records were screened based on the titles and abstracts, where the exclusion of irrelevant studies was implemented. Retrieval of the full-text articles was done. The articles successfully retrieved were assessed according to the appropriate tool for quality appraisal to minimize the risk of bias in this study.

Risk of Bias in Individual Studies

The full articles retrieved were assessed for quality assessment and risk of bias using tools depending on the study type: cohort studies, Newcastle Ottawa Scale (NOS); case reports, Joanna Briggs Institute (JBI) Critical Appraisal Checklist; animal studies, Systematic Review Centre for Laboratory animal Experimentation's (SYRCLE) risk of bias tool; and narrative reviews, Scale for the Assessment of Narrative Review Articles 2 (SANRA 2) [17-20]. The assessment tools differed in their criteria and passing scores, and a 70% score was required for each assessment tool to be accepted (Table 2).

**Table 2:** Quality assessment of each study type. *Maximum of two points are allotted in this category NOS, Newcastle Ottawa Scale; JBI, Joanna Briggs Institute; SANRA 2, Scale for the Assessment of Narrative Review Articles 2; SYRCLE, Systematic Review Centre for Laboratory animal Experimentation

Quality assessment tool	Type of study	Items and their characteristics	Total score	Accepted score >70%	Accepted studies	Number of accepted studies
SANRA 2 [17]	Narrative review	Six items: (1) justification of the article’s importance for the readership, (2) statement of concrete/specific aims or formulation of questions, (3) description of the literature search, (4) referencing, (5) scientific reasoning, and (6) appropriate presentation of data, scored as 0, 1, and 2	12	9	Doifode et al. [4], Aaldijk and Vermeiren [9], Liu et al. [21], Varesi et al. [22], Nandwana and Debbarma [23], Chen et al. [24], Wiatrak et al. [25], Chidambaram et al. [26]	8
NOS [18]	Cohort	Eight items: (1) representativeness of the exposed cohort, (2) selection of the nonexposed cohort, (3) ascertainment of exposure, (4) demonstration that outcome of interest was not present at the start of the study, (5) comparability of cohorts based on the design or analysis,* (6) assessment of outcome, (7) Was follow-up long enough for outcomes to occur? (8) Adequacy of follow-up of cohorts. Scoring was done by placing a point on each category. Scored as YES, NO, and NOT APPLICABLE.	9	7	Ling et al. [27]	1
SYRCLE [19]	Animal studies	Ten items: (1) Was the allocation sequence adequately generated and applied? (2) Were the groups similar at baseline, or were they adjusted for confounders in the analysis? (3) Was the allocation adequately concealed? (4) Were the animals randomly housed during the experiment? (5) Were the caregivers or investigators blinded from knowledge of which intervention each animal received during the experiment? (6) Were animals selected at random for the outcome assessment? (7) Was the outcome assessor blinded? (8) Were incomplete outcome data adequately addressed? (9) Are reports of the study free of selective outcome reporting? (10) Was the study free of other problems that could result in a high risk of bias? Scored as YES, NO, and UNCLEAR.	10	7	Sun et al. [28], Kim et al. [29]	2
JBI [20]	Case report	Eight items: (1) Clear description of the patient’s demographic characteristics, (2) description and presentation of the patient’s history as a timeline, (3) clear description of the patient’s current clinical condition on presentation, (4) clear description of diagnostic tests or methods and results, (5) clear description of the intervention(s) or treatment procedure(s), (6) clear description of the postintervention clinical condition, (7) identification and description of adverse events (harms) or unanticipated events, (8) takeaway lessons from the case report. Scored as YES, NO, UNCLEAR, or NOT APPLICABLE.	8	5.6	Hazan [30], Park et al. [31]	2

Data Collection, Items, and Analysis

As the topic of this study is still recent, this study is the first systematic review acknowledging this topic as per our knowledge. Furthermore, as we lack previous meta-analyses, systematic reviews, and human clinical trials, the first author has elected to include animal studies and the two published case studies regarding the FMT effect on AD.

Results 

Study Selection and Quality Assessment

**Figure 3:**
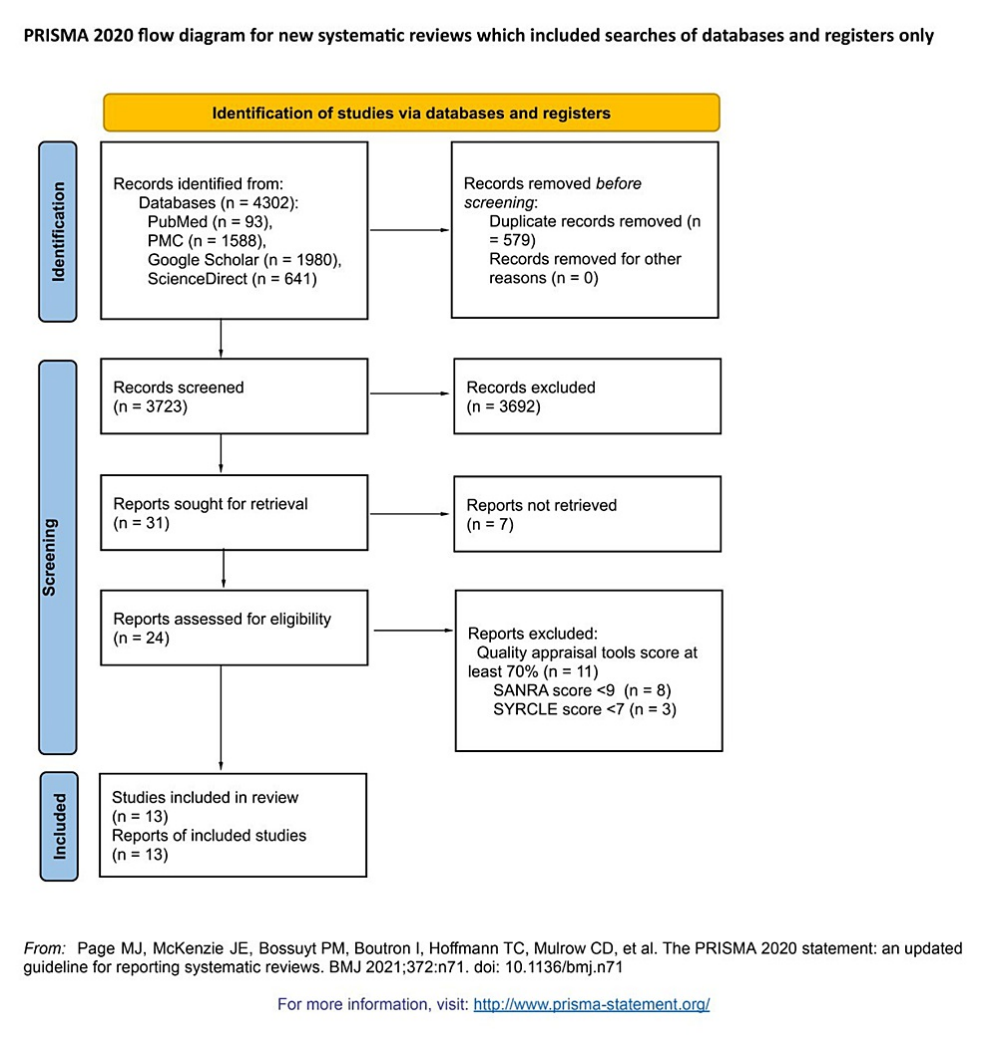
Flowchart of the study search selection. The PRISMA 2020 statement [16] PRISMA: Preferred Reporting Items for Systematic Reviews and Meta-Analyses; SANRA 2, Scale for the Assessment of Narrative Review Articles 2; SYRCLE, Systematic Review Centre for Laboratory animal Experimentation

At the start of the database search, there were 4,302 potentially relevant titles. After duplicates were removed, 3,723 records were retained. In the next step, 3,692 articles were removed while screening the records' titles and abstracts based on this review's PIO elements and eligibility criteria, leaving 31 articles for retrieval. Finally, a quality assessment for reports retrieved was conducted by the first author and reviewed and agreed upon by the second and third authors, yielding the 13 studies that scored more than 70% and were accepted in this study. These were eight narrative reviews, one cohort, two case studies, and two animal studies. A flow diagram showing the screening process and study selection is presented in Figure 3.

Tables 3-6 show how each study was evaluated according to the corresponding study type and the final result of the evaluation process. Table 3 demonstrates the scoring of narrative reviews using the SANRA 2 checklist based on six items.

**Table 3:** Results of the SANRA 2 assessment tool for narrative reviews by review authors. Passing score: 9/12 SANRA 2, Scale for the Assessment of Narrative Review Articles 2

First author, year	Justification of the article’s importance for the readership	Statement of concrete aims or formulation of the question	Description of the literature search	Referencing	Scientific reasoning	Appropriate presentation of data	Sum	Pass/Fail
Varesi et al., 2022 [22]	2	2	0	2	2	1	9	Pass
Aaldijk and Vermeiren, 2022 [9]	2	2	2	2	2	2	12	Pass
Cerovic et al., 2019 [1]	2	2	0	1	1	1	7	Fail
Chen et al., 2021 [24]	2	2	0	2	2	1	9	Pass
Chidambaram et al., 2021 [26]	2	2	0	2	2	1	9	Pass
Cho et al., 2020 [32]	2	2	0	2	1	1	8	Fail
Doifode et al., 2021 [4]	2	2	0	2	2	1	9	Pass
Fang et al., 2020 [33]	2	1	0	2	2	1	8	Fail
Ghaisas et al., 2015 [34]	2	2	0	2	1	1	8	Fail
Giridharan et al., 2022 [35]	2	2	0	2	1	1	8	Fail
Karout 2022 [36]	2	2	0	2	1	1	8	Fail
Liu et al., 2020 [21]	2	2	0	2	2	1	9	Pass
Nandwana et al., 2021 [23]	2	2	0	2	1	2	9	Pass
Wang et al., 2021 [37]	2	2	0	2	0	1	7	Fail
Wiatrak et al., 2022 [25]	2	2	0	2	1	2	9	Pass
Zhu et al., 2020 [38]	2	2	0	1	1	2	8	Fail

The JBI tool was used in assessing the two case reports in the review. Both of the studies have scored more than 70% (Table 4). 

**Table 4:** Results of the JBI assessment tool for case reports by review authors. Passing Score: 5.6/8 Y, yes; N, no; UN, unclear; N/A, not applicable; JBI, Joanna Briggs Institute

Author, year, record number	Demographic characteristics	Patient’s history as a timeline	Current clinical condition	Diagnostic/assessment methods	Intervention/treatment described	Postintervention clinical condition	Adverse events (harms) identified and described	Provide takeaway lessons	Result
Hazan, 2020 [30]	Y	Y	Y	Y	Y	Y	N	Y	Include
Park et al., 2021 [31]	Y	Y	Y	Y	Y	Y	Y	Y	Include

Table 5 demonstrates the NOS assessment tool for the only cohort study in this review.

**Table 5:** Results of the NOS assessment tool for observational studies by review authors. *Retrospective cohort study Passing score: 7/9 ✅, yes; ❎, no; N/A, not applicable, AD, Alzheimer's disease; NOS, Newcastle Ottawa Scale

Author, date	Representativeness of the exposed cohort	Selection of the nonexposed cohort	Ascertainment of exposure	Demonstration that the outcome of interest was not present at the start of the study	Comparability of the cohort based on design/analysis (2 points)	Assessment of the outcome	Was the follow-up long enough for the outcomes to occur	Adequacy of follow-up of cohorts	Pass/Fail
Ling et al., 2020 [27]	Truly representative of AD patients ✅	Drawn of same community ✅	Secure records (diagnosed based on the criteria of the National Institute of Neurological and Communicative Diseases and Stroke/AD and Related Disorders Association) ✅	N/A^*^	Study controls for fecal microbiota dysbiosis ✅	Record linkage ✅	❎	N/A^*^	Include

Finally, Table 6 demonstrates the assessment process of animal studies using the SYRCLE assessment tool.

**Table 6:** Results of the SYRCLE assessment tool for animal studies by review authors. Passing score: 70% ✅, yes; ❎, no; N/A, not applicable; SYRCLE, Systematic Review Centre for Laboratory animal Experimentation

Author	Sequence generation	Baseline characteristics	Allocation concealment	Random housing	Blinding	Random outcome assessment	Blinding	Incomplete outcome data	Selective outcome reporting	Other sources of bias	Include/exclude
Elangovan et al. [39]	❎	✅	✅ (allocated into groups rather than subjects)	N/A (not mentioned)	❎	N/A	❎	✅	✅	✅	Exclude
Kim et al. [29]	✅	✅	✅	✅	❎	N/A (All animals were assessed.)	❎	✅	✅	✅	Include
Sun et al. [28]	❎	✅	✅	✅	❎	N/A	❎	✅	✅	✅	Include
Wang et al. [40]	❎	✅	❎	✅	❎	N/A	❎	✅	✅	✅	Exclude
Zhang et al. [41]	❎	✅	❎	✅	❎	N/A	❎	✅	✅	✅	Exclude

Discussion 

Human Gut Microbiota and Dysbiosis

The microbes of the human gut exhibit a sizeable interindividual variability that is regularly explained by intrinsic, e.g., genetics, and extrinsic factors, e.g., diet, antibiotics, lifestyle, and illness [21]. As we get older, this microbiota undergoes dynamic changes, as proved by the fact that the diversity and number of gut microbiota decrease noticeably with aging [21,27]. In the study by Ling et al. in a Chinese population, 100 AD patients' samples were collected in addition to 71 age- and gender-matched, cognitively normal controls to investigate the functional and structural alterations of the fecal microbiota by targeting the V3-V4 region of the *16S rRNA* gene by MiSeq sequencing and to correlate their results with the clinical features. The data demonstrated a notable reduction in bacterial diversity along with changes in the taxonomic composition of the fecal microbiota of AD patients [27].

Dysbiosis, a phenomenon defined as the atypical changes in the content of gut flora, appears to be directly related to the pathophysiology of AD as it affects several distant organs [21]. For instance, two phyla seem more abundant in healthy individuals than others, *Bacteroidetes* and *Firmicutes*, which composite about 90% of the gut microbiota; the rest is composed of *Actinobacteria*, *Proteobacteria*, *Fusobacteria*, and *Verrucomicrobia* [23]. 

In patients with AD, studies have shown an increase in the number of *Escherichia/Shigella* along with a decrease in *Eubacterium rectale* and *Bacteroides fragilis* when comparing fecal samples of cognitively impaired patients with healthy controls using real-time quantitative polymerase chain reaction (qPCR) [24]. *B. fragilis* is an anti-inflammatory bacterium that can enhance the intestinal barrier and repair gut leakiness [24]. This may stand as one of the reasons why intestinal leakage is caused by a change in the gut microbiota along with bacterial components entering the brain. On the other hand, *Escherichia*, which promotes the body to maintain an inflammatory environment by inducing pro-inflammatory cytokines through an NLRP3-dependent mechanism, is generally upregulated in AD patients [24]. In addition, many bacteria have been reported to be increased in AD patients, such as *Prevotellaceae*, *Enterococcaceae*, *Ruminococcaceae*, *Lactobacillaceae*, *Dorea*, *Lactobacillus*, and *Streptococcus*.

In contrast, other types of bacteria have shown a decreased abundance such as *Negativicutes*, *Fusobacteriaceae*, *Eubacterium rectale*, *Veillonellaceae*, *Lanchnospiraceae*, and *Bacteroidaceae *[24]. This represents how different types of bacteria have varying effects on the brain and its cognitive function and explains the notion of gut dysbiosis as a potential target as a noninvasive biomarker to distinguish AD patients from the healthy population [27]. With that being said, studies breaking down the good vs. bad microbes are also essential in solidifying our understanding of the development and progression of AD.

Short-Chain Fatty Acids Part in AD and the Role of FMT in Restoring a Healthy Gut Microbiome

**Figure 4:**
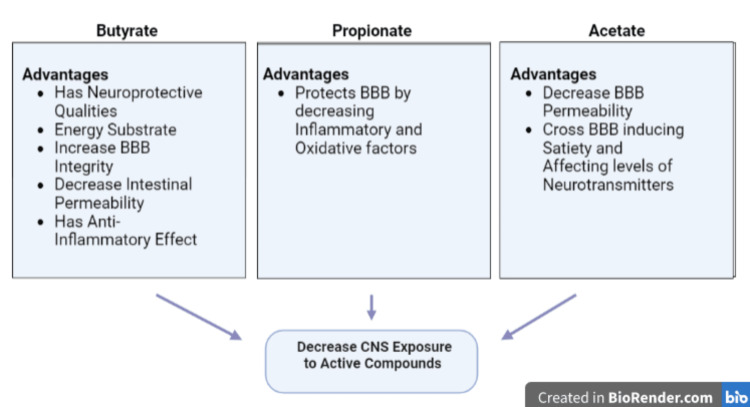
Types of short-chain fatty acids. BBB, blood-brain barrier; CNS, central nervous system Figure credit: BioRender.com

Bioactive products produced by bacteria explain how the gut microbiota modulates central physiological and pathological processes. Short-chain fatty acids (SCFAs), which are one of the metabolites coproduced by the host and its gut microbiota, influence host cells through diverse mechanisms and, therefore, are considered essential for host health with a pertinent role in AD prevention employing altering histone acetylation and cell proliferation, and activation of G-protein-coupled receptors [25,26]. SCFAs comprise butyrate, propionate, and acetate manufactured by *Clostridium*, *Eubacterium*, and *Butyrivibrio* bacteria [25]. Studies have shown butyrate to have neuroprotective qualities positively influencing the brain's functioning. It is also considered a significant energy substrate as it increases the mitochondrial production of adenosine triphosphate (ATP). In addition, it enforces BBB integrity while minimizing intestinal permeability and exhibiting an anti-inflammatory effect by inhibiting the immune system from secreting pro-inflammatory cytokines [25]. Sodium butyrate has positively affected AD mouse models, enhancing their learning and memory abilities, even in progressive and advanced stages of the disease [25]. Propionate, on the other hand, protects the BBB by decreasing the impact of pro-inflammatory and oxidative factors. Following their lead, acetate, another common SCFA, reduces the permeability of the BBB. This compound effect decreases the exposure of the CNS to active compounds originating from outside the CNS. In addition, acetate can cross the BBB, inducing a satiety feeling and causing a change in the neurotransmitters' levels (Figure 4) [25].

Thus, as is reasonable and expected, restoration of optimal levels of SCFAs and a healthy gut microbiome have been shown by multiple studies to reduce neurodegenerative pathogenesis. In particular, SCFAs have disrupted the assembly of Aβ oligomers in AD into neurotoxic aggregates by meddling with their protein-protein interactions with Aβ peptides [28].

Furthermore, a recent study using APPswe/PS1dE9 transgenic (Tg) mouse model revealed that FMT could lessen the brain deposition of Aβ and the phosphorylation of Tau protein along with the levels of Aβ40 and Aβ42 [28]. Besides, cognitive function refinement and synaptic plasticity rise have also been noted in Tg mice [23, 28]. Accordingly, the FMT effect on restoring such active mediators may serve as a future therapeutic target for neurodegenerative disorders, particularly AD.

FMT in Animal and Human Studies

FMT has already been used successfully to treat *C. difficile* infections in humans [12]. However, due to technical and ethical concerns, FMT has not been implemented and used routinely in clinical practice in neurological diseases; it is still restricted in animal models' scientific studies [24]. Nevertheless, these initial studies have shown full of promise, though not irrefutable, results [22]. Table 7 shows two of these animal studies in detail.

**Table 7:** Murine studies of FMT in AD subjects. AD, Alzheimer's disease; FMT, fecal microbiota transplantation; *n*, number; 5×FAD, 5× familial Alzheimer's disease; C57BL/6, C57 black mice; BDNF, brain-derived neurotrophic factor; TNF, tumor necrosis factor; IL, interleukin; WT, wild type; APPswe/PS1dE9, APP/PS1-21; MWM, Morris water maze test; ORT, object recognition test; PSD-95, postsynaptic density protein 95; COX2, cyclooxygenase 2; CD11b, integrin alpha M chain; SCFAs, short-chain fatty acids; ↑, increase; ↓, decrease.

Reference	Study cohort/sample size	Donor	Recipient	Transplantation technique	Results
Kim et al. [29]	Mice (*n* = 8)	5×FAD mice	C57BL/6 mice	Oral gavage (200 mL for five consecutive days)	↓ Adult hippocampal neurogenesis and BDNF expression ↑ p21 expression ↑ Microglia activation ↑ TNF-α and IL-1β ↑ Colon and plasma pro-inflammatory cytokines
Sun et al. [28]	Mice (*n* = 8)	WT mice	APPswe/PS1dE9 transgenic (Tg) mouse model	Intragastrically (0.2 mL of the fresh fecal solution once daily for four weeks)	↑ Cognitive function (MWM and ORT tests) ↓ Amyloid-beta brain deposition (Aβ40 and Aβ42) ↓ Tau protein phosphorylation ↑ Synaptic plasticity (increased PSD-95 and synapsin I) ↓ COX2 and CD11b ↑ SCFA and microbiota composition

Regarding human studies, two case studies have been reported so far that show quite encouraging results [30,31]. Hazan et al. Reported an improvement in AD symptoms (mood, memory, and cognitive function) in an 82-year-old male after FMT from his wife, an 85-year-old, and his symptoms resolved afterward within two months. In addition, the Mini-Mental State Examination (MMSE) score of the FMT recipient has increased from 20, which is mild cognitive impairment, to 26, showing normal cognitive function two months after the transplant. More improvement was seen in the four months posttransplant follow-up with an MMSE score of 29 [30]. The second case study involved a 90-year-old woman (the recipient) with AD and severe *C. difficile* infection who received FMT twice from a 27-year-old healthy male. In this case, an improvement in cognitive function, microbiota diversity, and SCFAs production were also observed [31].

Limitations

This review only included studies published in the English language, using four databases from 2012 to 2022. Most of the evidence in this review referred to animal studies due to the lack of clinical trials on human objects, further limiting our findings. In addition, only full free-text articles were acquired, and thus, this may have precluded some of the eligible studies.

## Conclusions

In conclusion, FMT can potentially become one of the modalities in treating AD, exerting its effect through the microbiota-gut-brain axis. The diversity of the gut microbiota in AD patients is widely changed compared with the healthy population as the types of bacteria abundant in healthy people differ from those in AD patients. This is speculated to participate in AD pathophysiology because the original gut bacteria metabolize peptides, soluble fibers, and dietary proteins, bringing out SCFAs and tryptophan along with other metabolites. These products work to lessen gut inflammation along with BBB permeability. Animal studies have demonstrated the FMT effect to restore the SCFAs and a healthy microbiome to disrupt the Aβ oligomers, decreasing AD's pathogenesis. However, the lack of clinical trials due to ethical concerns has hindered our findings in humans.

Nevertheless, two case reports were identified in which 82- and 90-year-old patients had received FMT from fit donors and the recipients exhibited cognition improvements. Future recommendations from this review would be to conduct further studies, especially randomized controlled trials, where FMT is implemented in patients of AD at various stages of the disease, following them up for longer durations. Thus, a further detailed assessment of its effect as a possible AD intervention could be evaluated.
